# Clinical and Radiological Presentations of Various Pulmonary Infections in Hospitalized Diabetes Mellitus Patients: A Prospective, Hospital-Based, Comparative, Case Series Study

**DOI:** 10.1155/2021/8878746

**Published:** 2021-03-20

**Authors:** Pravesh Vishwakarma, Kauser Usman, Rajiv Garg, Jyoti Bajpai, Rishi Sethi, Akshyaya Pradhan

**Affiliations:** ^1^Department of Cardiology, King George's Medical University, Lucknow, Uttar Pradesh 226003, India; ^2^Department of Internal Medicine, King George's Medical University, Lucknow, Uttar Pradesh 226003, India; ^3^Department of Respiratory Medicine, King George's Medical University, Lucknow, Uttar Pradesh 226003, India

## Abstract

**Background:**

Diabetes mellitus is associated with increased rate of respiratory tract infections. The objective was to compare demographic, clinical, serum biochemical, and typical and atypical radiological profiles among hospitalized diabetics and nondiabetics with lower respiratory tract infection. *Material and Methods*. A prospective, hospital-based, consecutive, comparative observational study of 12-month study duration was conducted. Patients aged 13–90 years diagnosed with lower respiratory tract infection with or without diagnosed diabetes mellitus participated in the study. Demographic, clinical, serum biochemistry, and radiological profiles of diabetics (*n* = 44) and nondiabetics (*n* = 53) were compared.

**Results:**

Diabetics were older than nondiabetics at presentation (*p* < 0.0001). Difference in mean random blood sugar (RBS) (*p* < 0.001), fasting blood sugar (FBS) (*p* < 0.001), and postprandial blood sugar (PPBS) (*p* < 0.0001) was significant between diabetics and nondiabetics. Nondiabetics more frequently presented with fever (*p* = 0.0032), chest pain (*p* = 0.0002), and hemoptysis (*p* = 0.01) as compared to diabetics. Diabetics more frequently presented with extreme temperatures (hypothermia or hyperpyrexia) (*p* = 0.022), lower serum sodium levels (*p* = 0.047), and lower partial arterial pressure (*p* < 0.001) than nondiabetics. The mean pneumonia patient outcomes research team (PORT) risk score was higher in diabetics (124.84 ± 41.31) compared to nondiabetics (77.85 ± 39.77) (*p* < 0.001). Diabetics more commonly displayed bilateral lesions with multilobe or lower lobe involvement, the most common type of lesion being exudative.

**Conclusion:**

Diabetic patients usually had severe pulmonary infection and poor prognosis as suggested by higher mean PORT risk score. They also more frequently presented with bilateral lesions with multilobe or lower lobe involvement as evidenced by radiography as compared to nondiabetic patients.

## 1. Introduction

Diabetes mellitus is a vastly prevalent chronic metabolic disorder escalating at an alarming rate around the globe. India has contributed substantially to this global burden. Prevalence of diabetes in India increased from 26 million in 1990 to 65 million in 2016 [1]. This metabolic disorder is identified by hyperglycemia resulting from reduced insulin secretion, reduced glucose usage, and increased glucose production [2, 3]. The hyperglycemic state, lowered immunity, pulmonary microangiopathy, and pulmonary dysfunction are frequently accompanied by sequelae such as severe hospital-acquired pneumonia, severe pulmonary infections with antimicrobial resistance, and chronic complications such as renal failure, coronary disease, diabetic nephropathy, and diabetic retinopathy [4].

The lung is a target organ affected by diabetes mellitus—pulmonary and vascular functions are closely associated. Acute and chronic respiratory infections such as tuberculosis and pneumonia as well as other rare infectious diseases are frequently observed in diabetic patients [5]. Pulmonary infections are one of the most frequently encountered infections in diabetic patients. Several studies have revealed that pulmonary infections in diabetics predispose this subset of patients to more severe clinical manifestations, longer duration of diabetes, longer duration of treatment, more frequent complications, and increased mortality [2, 5–10]. Moreover, certain types of pulmonary infections are more prevalent among diabetics than nondiabetics. The clinical spectrum and radiological presentation of such infections differ from those of nondiabetics. Further investigations are warranted to determine the pattern of infection in these patients. Against this background, we conducted the present study with the objective to compare demographic, clinical, serum biochemical, and typical and atypical radiological profiles among hospitalized diabetics and nondiabetics with lower respiratory tract infection.

## 2. Materials and Methods

### 2.1. Study Design and Patient Population

A prospective, hospital-based, consecutive, comparative, case series study comparing diabetics and nondiabetics with lower respiratory tract infection was conducted at a tertiary care centre King George's Medical University, U.P., Lucknow, in India over a 12-month period from September 2007 to August 2008. All patients aged 13–90 years admitted in inpatient wards of Gandhi Memorial and Associated Hospitals (Department of Internal Medicine and Department of Pulmonary Medicine), King George's Medical University, U.P., Lucknow, with diagnosis of lower respiratory tract infection with or without evidence of diabetes mellitus were included in the study.

Patients who (i) had died within 8 hours of admission, (ii) could not be investigated, (iii) had impaired fasting plasma glucose or impaired glucose tolerance, (iv) had malignancy, or (v) refused to provide written informed consent were excluded from the study. The study was approved by the Institutional Ethics Committee prior to the start of the study. Written informed consent to use the patient's clinical records for scientific purposes was obtained from each patient before study enrolment.

### 2.2. Data Collection for All Study Patients

After explaining the study and obtaining written informed consent, all patients provided a complete clinical history and underwent a thorough physical examination. Patient demographics such as age and gender and presenting symptoms such as fever, cough, expectoration, hemoptysis, chest pain, and dyspnea were recorded. During the physical examination, hypotension (systolic blood pressure < 90 mmHg or use of vasopressor), tachycardia (pulse rate > 110/min), tachypnea (respiratory rate > 30/min), hyperpyrexia (temperature > 104°F), or hypothermia (temperature < 95°F) was recorded. A battery of basic biochemical and hematological tests was performed for all patients. Similarly, chest radiographs for all patients were obtained.

### 2.3. Additional Data Collection for Diabetic Patients

Patients with initial blood glucose levels or a prior diagnosis of diabetes provided a detailed history including duration of diagnosed diabetes mellitus and ongoing treatment. Glycemic monitoring (fasting and postprandial blood sugar) along with measurement of hemoglobin A_1c_ (HbA_1c_) was performed at the time of admission to determine the level of glycemic control. In addition, the presence of diabetes-related complications was investigated by evaluating patient hospital records, current clinical manifestations, blood or urine biochemistry abnormalities, and when necessitated specific tests.

### 2.4. Lower Respiratory Tract Infection

Lower respiratory tract infection was diagnosed by the presence of ≥2 of the following symptoms: (i) fever, (ii) new or increasing cough or sputum production, (iii) dyspnea, (iv) chest pain, or (v) new focal signs on chest examination in addition to the presence of at least one opacity on chest radiography consistent with infectious pathology and/or isolation of suspected microorganism from sputum, pleural fluid, or blood. The validated pneumonia severity index (PSI)/pneumonia patient outcomes research team (PORT) score [11, 12] was used to categorize risk class of the patient at the time of admission [13].

### 2.5. Diabetes Mellitus

Diabetes mellitus was diagnosed according to the standard American Diabetic Association guidelines [14]. The criteria for diagnosis are as follows: (i) symptoms of hyperglycemia and random plasma glucose ≥ 200 mg/dL; random was defined as any time of day regardless of time since last meal; the classic symptoms of hyperglycemia include polyuria, polydipsia, and unexplained weight loss; (ii) fasting plasma glucose ≥ 126 mg/dL; fasting was defined as no calorie intake for at least 8 hours; or (iii) 2-hour plasma glucose ≥ 200 mg/dL during an oral glucose tolerance test. The test was performed as described by the World Health Organization using a glucose load containing an equivalent of 75 g anhydrous glucose dissolved in water.

### 2.6. Impaired Fasting Glucose and Impaired Glucose Tolerance

Patients were diagnosed with impaired fasting glucose or impaired glucose tolerance by fasting glucose ≥ 100 mg/dL but <126 mg/dL or 2-hour values in the oral glucose tolerance test (OGTT) of >140 mg/dL but <200 mg/dL. All cases in the present study were subjected to the oxidase method to avoid interference from lipid, bilirubin, uric acid, and antidiabetic drugs.

### 2.7. Assessment of Glycemic Control

Assessment of glycemic control, i.e., differentiation of diabetic patients into controlled or uncontrolled, was done by measuring HbA_1c_ as outlined by the American Diabetic Association [14]. Patients with glycated HbA_1c_levels < 7% were considered to have controlled diabetes mellitus, while patients with levels beyond this were considered to have uncontrolled diabetes mellitus.

### 2.8. Chest Radiography

Posterior-anterior chest X-ray views were taken in all patients, and if required, lateral views were also taken. Lesions were described according to (i) site, (ii) zone of involvement, or (iii) nature. Sites of the lesion were either unilateral (right or left) or bilateral. Zones of involvement included upper, middle, or lower zones. The upper zone lied above the right anterior border of the second rib. The middle zone lied between the right anterior border of the second and fourth ribs. The lower zone lied between the right anterior border of the fourth rib and diaphragm. The lesions were categorized as exudative, consolidation, pleural effusion, cavitation, pneumothorax, or hydropneumothorax. Exudative lesions were lesions with predominantly small shadows merging with each other to produce shadows resembling cotton wool or clone-like in appearances. Consolidation lesions were patchy, opaque, exudative lesions of segmental or lobar distribution with homogenous density. Pleural effusions were identified as homogenous, dense, and opaque effusions with ill-defined upper limits that were laterally high with filling of costophrenic angle. Cavitations were single or multiple, clear-cut, homogenous, low-density cavities filled with air. Pneumothorax was defined as an area of hyperluscency with absence of bronchovascular markings and presence of collapsed lung margins. The pleural cavity is filled with air. Hydropneumothorax was defined as the presence of both air and fluid in the pleural space.

### 2.9. Statistical Analysis

Continuous variables are presented as mean ± standard deviation, while categorical variables are presented as frequency and percentages. Continuous variables were compared using the Student *t*-test. Categorical variables were compared using either the chi-square test or Fisher exact test. A *p* value < 0.05 was considered statistically significant. The statistical evaluation of data was done using the Statistical Package for the Social Sciences (SPSS; Chicago, IL, USA) program, version 14.

## 3. Results

**Figure 1:**
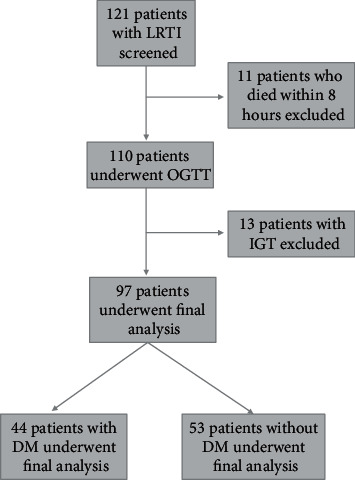
Flow diagram of the study.

A total of 121 patients fulfilled the study inclusion criteria and were enrolled in the study. Out of these 121 patients, 11 patients died within 8 hours of hospitalization and could not be further investigated. These patients were excluded from the study analysis. Of the 110 patients with lower respiratory tract infections, 44 (40.0%) patients had blood sugar levels within diabetic range on OGTT (4 of which were diagnosed during hospital stay), 53 (48.2%) had blood sugar levels within normal range, and 13 (11.8%) had impaired glucose tolerance and were therefore excluded from the study. Thus, 97 patients constituted the study population. 44 (40.0%) patients were diagnosed as diabetic and were designated as cases and the remainder 53 (48.2%) patients were diagnosed as nondiabetic and were designated as controls. The study flow is illustrated in Figure 1.

### 3.1. Demographic Profile of Diabetics and Nondiabetics with Pulmonary Infections

The mean age of diabetics and nondiabetics with pulmonary infection was 54.73 ± 12.18 and 41.15 ± 18.77 years, respectively. Diabetics were significantly older than nondiabetics (*p* < 0.0001). The study population with pulmonary infection was predominantly middle aged (30-60 years; 66% and 49% in case and control groups, respectively). Males comprised of 32 patients in (72.7%) diabetics and 35 (66.0%) nondiabetics, respectively (*p* = 0.48). The demographic profile of diabetics and nondiabetics with pulmonary infections is demonstrated in Table 1.

### 3.2. Diabetic Profile of Pulmonary Infection Patients

**Figure 2:**
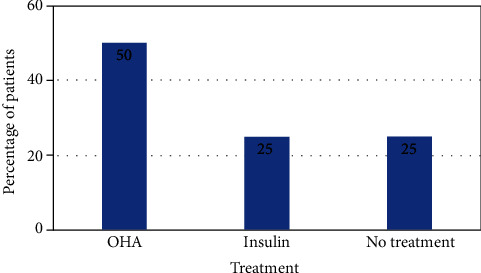
Prehospital treatment profile for patients with diabetes mellitus in the study. OHA: oral hypoglycemic agent.

The mean duration of diabetes in patients with pulmonary infection was found to be 6.23 ± 5.01 years. Orally administered antihyperglycemic agents were the most prescribed treatment used by half of the diabetics. A quarter of the diabetics used insulin, while the other quarter of patients was not adhering to any treatment. Prehospital treatment profile of the diabetic population is displayed in Figure 2. The difference in mean random blood sugar (RBS) (*p* < 0.001), fasting blood sugar (FBS) (*p* < 0.001), and postprandial blood sugar (PPBS) (*p* < 0.0001) between diabetics and nondiabetics was statistically significant. However, the difference in mean RBS (*p* = 0.20), FBS (*p* = 0.20), and PPBS (*p* = 0.05) between uncontrolled and controlled diabetic patients was not statistically significant, although these values were lower in controlled diabetics. The blood sugar levels of these groups are detailed in Table 2. Of the 44 diabetics, 16 (36.4%) patients had controlled diabetes, while 28 (63.6%) had uncontrolled diabetes. Twenty (45.5%) diabetic patients had albuminuria > 300 mg/day, 10 (22.7%) diabetics had albuminuria < 300 mg/day, and 14 (31.8%) diabetics did not have albuminuria. Nephropathy and retinopathy were the most common complications observed in 22 (50.0%) and 19 (43.2%) diabetics, respectively. HbA_1c_ levels, albuminuria levels, and complications of diabetes are described in Table 3.

### 3.3. Clinical Profile of Pulmonary Infection in Diabetic and Nondiabetic Patients

**Figure 3:**
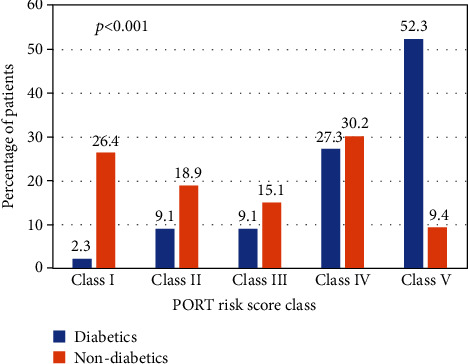
PORT risk score classes of diabetics and nondiabetics with pulmonary infections in the study. PORT: patient outcomes research team.

Nondiabetics more frequently presented with fever (*p* = 0.0032), chest pain (*p* = 0.0002), and hemoptysis (*p* = 0.01) as compared to diabetics. Diabetics had more comorbidities than nondiabetics (*p* = 0.021). Diabetics more frequently presented with extreme temperatures (hypothermia or hyperpyrexia) (*p* = 0.022), lower serum sodium levels (*p* = 0.047), and lower partial arterial pressure (*p* < 0.001) than nondiabetics. The mean PORT risk score was higher in diabetics (124.84 ± 41.31) compared to nondiabetics (77.85 ± 39.77) (*p* < 0.001). Majority of diabetic patients fell into PORT score class IV and V, while the majority nondiabetic patients fell into classes I–III (*p* < 0.001). The clinical profile of pulmonary infections in diabetics and nondiabetics is demonstrated in Table 4, and PORT score class of diabetics and nondiabetics is illustrated in Figure 3.

### 3.4. Radiological Profile of Pulmonary Infections in Diabetic and Nondiabetic Groups

**Figure 4:**
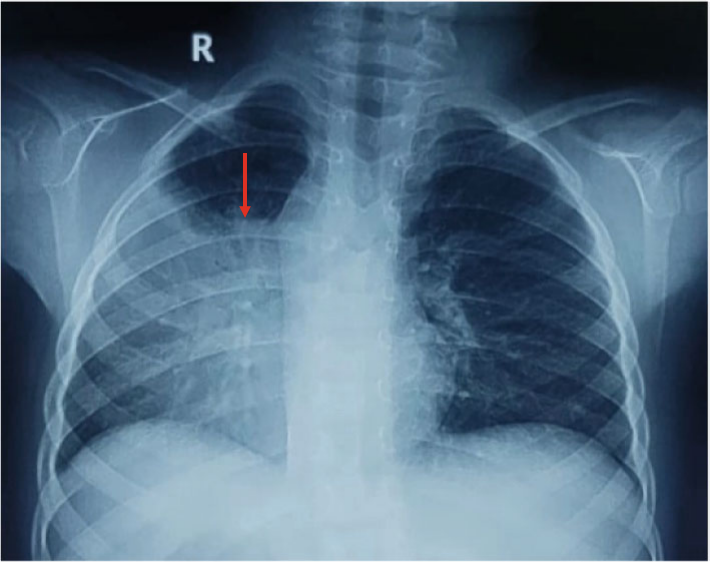
Chest X-ray showed right side mid and lower zone consolidation in nondiabetic patients.

**Figure 5:**
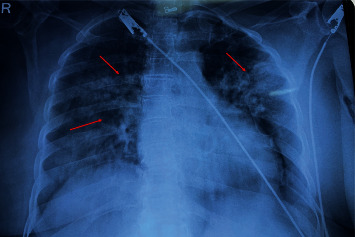
Chest X-ray showed bilateral multifocal mid and lower zone infiltrates/exudates in diabetic patients.

Unilateral right- and left-sided lesions were more prevalent in nondiabetics (Figure 4), while bilateral lesions were more prevalent in diabetics (Figure 5) (*p* = 0.022). Upper, middle, lower, and multilobe involvement was observed in 10 (22.7%), 2 (4.5%), 16 (36.4%), and 13 (29.5%) diabetics and 12 (22.6%), 7 (13.2%), 18 (34.0%), and 8 (15.1%) nondiabetics, respectively, although this finding was not statistically significant. The radiological profile of pulmonary infections in diabetic and nondiabetics is detailed in Table 5.

## 4. Discussion

Diabetes mellitus has evidenced to consequence unfavorable outcomes in patients with respiratory tract infections. Several earlier studies have investigated the frequency and pattern of respiratory infections in both diabetic and nondiabetics. Saibal et al. [3] reported a mean age of 56.3 ± 12.2 and 35.7 ± 10.5 years in diabetics and nondiabetics with community-acquired pneumonia, respectively. Wang et al. [15] observed a mean age of 60.8 and 59.1 years in diabetics and nondiabetics with pulmonary tuberculosis, respectively. Fernández et al. [16] found a median age of 76.1 and 60.1 years in diabetics and nondiabetics with community-acquired pneumonia, respectively. Park et al. [17] observed a median age of 66.0 and 42.0 years in uncontrolled diabetics and nondiabetics with pulmonary tuberculosis, respectively. Similarly, Alisjahbana et al. [18] revealed a median age of 45.0 and 27.0 years in diabetics and nondiabetics with pulmonary tuberculosis, respectively. In line with these findings, the present study revealed a mean age of 54.73 ± 12.18 and 41.15 ± 18.77 years in diabetics and nondiabetics with lower respiratory infections, respectively. These findings reveal a trend of higher mean age in diabetics than nondiabetics with respiratory infections [2]. This trend may be explained by greater prevalence of comorbidities in diabetic patients above the age of 60 years. Due to these comorbidities and weakening immune system parallel to increase in age, diabetics become more prone to various infections [16].

Symptomatology did not differ between diabetics and nondiabetics with lower respiratory infections. The most common clinical presentations were fever, cough, and expectoration in the present study. Similarly, in the study by Chandra et al. [6], the most common clinical manifestations were fever, cough, and shortness of breath. Moreover, in the study by Alisjahbana et al. [18], cough, weight loss, and fever were the common symptoms. Wang et al. [15] found higher presentation with fever and hemoptysis in diabetic patients with pulmonary tuberculosis. Interestingly, Saibal et al. [3] revealed altered mental status and hypotension as the predominant clinical features in diabetic patients with community-acquired pneumonia.

The PSI/PORT score is a validated tool used as a clinical predictor to stratify patients according to the severity of community-acquired pneumonia. The present study revealed that the majority of patients with diabetes with lower respiratory infections fell into classes IV and V, while contrastingly nondiabetics fell into classes I-III. CURB-65 is another risk stratification tool used to stratify patients with community-acquired pneumonia. The study by Saibal et al. [3] revealed higher CURB-65 score in diabetics than nondiabetics. These findings evidence a higher clinical risk for diabetics than nondiabetics, regardless of the tools used for risk stratification.

Diabetics are more prone to unusual radiographic presentation. Lesions with predominant bilateral [9], lower [6, 15, 19], and multilobe involvement [3, 6, 17] with higher incidences of pleural effusion [3, 6, 15] have been identified in diabetics. In the present study, the radiological presentation of diabetics was characteristic of bilateral, lower, and multilobe involvement with exudative and consolidative lesions. This difference in these clinical manifestations in diabetics and nondiabetics may be explained by altered capillary permeability, less vigorous and more vulnerable immune systems, and altered neutrophil and macrophage function observed in diabetics [3]. Other authors have asserted these disparities may be due to demography and patient selection process [15]. The effect of diabetes mellitus on the radiological presentation of pulmonary tuberculosis is critical because misinterpretations may hinder appropriate diagnosis and treatment.

### 4.1. Study Limitations

The study has a few limitations. The first is the small sample size and short duration of the study period. Secondly, we did not adjust for possible confounding factors.

## 5. Conclusion

Diabetic patients usually had severe pulmonary infection and poor prognosis as suggested by higher mean PORT risk score. They also more frequently presented with bilateral lesions with multilobe or lower lobe involvement as evidenced by radiography as compared to nondiabetic patients.

## Figures and Tables

**Table 1:** Demographic profile of diabetics and nondiabetics with pulmonary infections.

Variable	Diabetics (*n* = 44)	Nondiabetics (*n* = 53)	*p* value
Age (years) (mean ± SD)	54.73 ± 12.18	41.15 ± 18.77	<0.0001
Age, *n* (%)			
<30 years	0 (0%)	19 (35.8%)	<0.0001
30–60 years	29 (65.9%)	26 (49.1%)	
>60 years	15 (34.1%)	8 (15.1%)	
Males, *n* (%)	32 (72.7%)	35 (66.0%)	0.4

**Table 2:** Blood sugar levels of diabetics versus nondiabetics and uncontrolled diabetes mellitus versus controlled diabetes mellitus.

Variable	Diabetics (*n* = 44)	Nondiabetics (*n* = 53)	*p* value	Uncontrolled diabetes mellitus (*n* = 28)	Controlled diabetes mellitus (*n* = 16)	*p* value
RBS (mg/dL)	189.84 ± 91.34	101.08 ± 21.05	<0.001	206.36 ± 95.15	167.81 ± 78.00	0.20
FBS (mg/dL)	189.25 ± 85.62	101.02 ± 37.36	<0.001	201.5 ± 88.67	167.81 ± 78.07	0.20
PPBS (mg/dL)	275.76 ± 29.83	133.25 ± 29.83	<0.0001	298.37 ± 113.08	262.88 ± 89.14	0.05

Data are expressed as mean ± standard deviation. RBS: random blood sugar; FBS: fasting blood sugar; PPBS: postprandial blood sugar.

**Table 3:** HbA_1c_ levels, albuminuria levels, and complications of diabetes.

Variable	Diabetics (*n* = 44)
HbA_1c_, *n* (%)	
<7% (controlled)	16 (36.4%)
>7% (uncontrolled)	28 (63.6%)
Albuminuria, *n* (%)	
>300 mg/day	20 (45.5%)
<300 mg/day	10 (22.7%)
Nil	14 (31.8%)
Complications of diabetes	
Nephropathy, *n* (%)	22 (50.0%)
Retinopathy, *n* (%)	19 (43.2%)
Neuropathy, *n* (%)	3 (6.8%)
None, *n* (%)	16 (36.4%)

**Table 4:** Clinical profile of pulmonary infections in diabetics and nondiabetics.

Variable	Diabetics (*n* = 44)	Nondiabetics (*n* = 53)	*p* value
Symptoms			
Fever, *n* (%)	28 (63.6%)	47 (88.7%)	0.0032
Cough, *n* (%)	32 (72.7%)	44 (83.0%)	0.22
Expectoration, *n* (%)	21 (47.7%)	26 (49.1%)	0.90
Chest pain, *n* (%)	5 (11.4%)	24 (45.3%)	0.0002
Hemoptysis, *n* (%)	9 (20.5%)	24 (45.3%)	0.01
Dyspnea, *n* (%)	26 (59.1%)	31 (58.5%)	0.88
Conscious	36 (81.8%)	50 (94.4%)	0.053
Unconscious	8 (18.2%)	3 (5.7%)	
Associated comorbidities			
Heart failure, *n* (%)	3 (6.8%)	0 (0%)	0.021
Renal failure, *n* (%)	7 (15.9%)	1 (1.9%)	
Chronic liver disease, *n* (%)	0 (0%)	1 (1.9%)	
None, *n* (%)	37 (84.1%)	51 (96.2%)	
Systolic blood pressure, *n* (%)			
≤90 mmHg	6 (13.6%)	2 (3.8%)	0.08
>90 mmHg	38 (686.4%)	51 (96.2%)	
Pulse rate, *n* (%)			
≥100/min	19 (43.2%)	25 (47.2%)	0.69
<100/min	25 (56.8%)	28 (52.8%)	
Respiratory rate, *n* (%)			
≥30/min	19 (43.2%)	21 (39.6%)	0.72
<30/min	25 (56.8%)	32 (60.4%)	
Temperature, *n* (%)			
<95°F	7 (15.9%)	1 (1.9%)	0.022
94–104°F	36 (81.8%)	52 (98.1%)	
>104°F	1 (2.3%)	0 (0%)	
TLC, *n* (%)			
≥12,000 per cmm	20 (45.5%)	34 (64.2%)	0.064
<12,000 per cmm	24 (54.5%)	19 (35.8%)	
Serum sodium, *n* (%)			
<130 mEq/dL	13 (29.5%)	7 (13.2%)	0.047
≥130 mEq/dL	31 (70.5%)	46 (86.8%)	
Blood urea, *n* (%)			
≥60 mg/dL	8 (18.2%)	5 (9.4%)	0.2
<60 mg/dL	36 (81.8%)	48 (90.6%)	
Arterial pH, *n* (%)			
<7.35	20 (45.5%)	17 (32.1%)	0.18
>7.35	24 (54.5%)	36 (67.9%)	
pO2			
<60 mmHg	25 (56.8%)	51 (96.2%)	<0.001
≤60 mmHg	19 (43.2%)	2 (3.8%)	
PORT risk score (mean ± SD)	124.84 ± 41.31	77.85 ± 39.77	<0.001
Tubercular, *n* (%)	15 (34.1%)	21 (39.6%)	0.57
Nontubercular, *n* (%)	29 (65.9%)	32 (60.4%)	
Sputum microscopy, *n* (%)			
AFB positive	9 (20.5%)	16 (30.2%)	0.50
AFB negative	6 (13.6%)	5 (9.4%)	
Mantoux test, *n* (%)			
Positive	8 (18.2%)	10 (18.9%)	0.99
Negative	7 (15.9%)	10 (18.9%)	

TLC: total leukocyte count; pO2: partial pressure of oxygen; AFB: acid-fast bacteria; PORT: pneumonia patient outcomes research team.

**Table 5:** Radiological profile of pulmonary infections in diabetics and nondiabetics.

	Diabetics (*n* = 44)	Nondiabetics (*n* = 53)	*p* value
Site of lesion			
Unilateral (right), *n* (%)	16 (36.4%)	29 (54.7%)	0.022
Unilateral (left), *n* (%)	9 (20.5%)	14 (26.4%)	
Bilateral, *n* (%)	19 (43.2%)	10 (18.9%)	
Zone of lesion			
Upper, *n* (%)	10 (22.7%)	12 (22.6%)	0.68
Middle, *n* (%)	2 (4.5%)	7 (13.2%)	
Lower, *n* (%)	16 (36.4%)	18 (34.0%)	
Multilobed, *n* (%)	13 (29.5%)	8 (15.1%)	
Nature of lesion			
Exudative, *n* (%)	20 (45.5%)	14 (26.4%)	0.34
Consolidation, *n* (%)	16 (36.4%)	24 (45.3%)	
Milliary, *n* (%)	2 (4.5%)	2 (3.8%)	
Fibrocavitary, *n* (%)	5 (11.4%)	3 (5.7%)	
Pleural, *n* (%)	11 (25.0%)	18 (34.0%)	

## Data Availability

The data is available with the first author and will be available on request.
